# Impact of Sprouting Process on the Protein Quality of Yellow and Red Quinoa (*Chenopodium quinoa*)

**DOI:** 10.3390/molecules29020404

**Published:** 2024-01-14

**Authors:** Hassan Barakat, Maryam M. Al-Qabba, Raya Algonaiman, Khadija S. Radhi, Abdulkarim S. Almutairi, Muath M. Al Zhrani, Ahmed Mohamed

**Affiliations:** 1 Department of Food Science and Human Nutrition, College of Agriculture and Veterinary Medicine, Qassim University, Buraydah 51452, Saudi Arabia; 2 Department of Food Technology, Faculty of Agriculture, Benha University, Moshtohor 13736, Egypt; 3 Maternity and Children Hospital, Qassim Health Cluster, Ministry of Health, Buraydah 52384, Saudi Arabia; mmalqabi@moh.gov.sa; 4 Department of Food Science and Nutrition, College of Sciences, Taif University, P.O. Box 11099, Taif 21944, Saudi Arabia; ksradhi@tu.edu.sa; 5 Al Rass General Hospital, Qassim Health Cluster, Ministry of Health, Ibn Sina Street, King Khalid District, Al Rass 58883, Saudi Arabia; abdulkarimsa@moh.gov.sa; 6 Department of Applied Medical Science, College of Applied, Bishah University, Bishah 67714, Saudi Arabia; muath@ub.edu.sa; 7 Department of Biochemistry, Faculty of Agriculture, Benha University, Moshtohor 13736, Egypt; ahmed.mohamed@fagr.bu.edu.eg

**Keywords:** quinoa, germination, protein, nutritional quality, nutrition, amino acids

## Abstract

The demand for plant-based proteins has increased remarkably over the last decade. Expanding the availability and variety of plant-based protein options has shown positive potential. This study aimed to investigate the qualitative and quantitative changes in amino acids of yellow and red quinoa seeds (YQ and RQ) during a 9-day germination period. The results showed that the germination process led to an increase in the total amino acids by 7.43% and 14.36% in the YQ and RQ, respectively. Both varieties exhibited significant (*p* < 0.05) increases in non-essential and essential amino acids, including lysine, phenylalanine, threonine, and tyrosine. The content of non-essential amino acids nearly reached the standard values found in chicken eggs. These results were likely attributed to the impact of the germination process in increasing enzymes activity and decreasing anti-nutrient content (e.g., saponins). A linear relationship between increased seeds’ hydration and decreased saponins content was observed, indicating the effect of water absorption in changing the chemical composition of the plant. Both sprouts showed positive germination progression; however, the sprouted RQ showed a higher germination rate than the YQ (57.67% vs. 43.33%, respectively). Overall, this study demonstrates that germination is a promising technique for enhancing the nutritional value of quinoa seeds, delivering sprouted quinoa seeds as a highly recommended source of high-protein grains with notable functional properties.

## 1. Introduction

In the past decade, plant-based proteins have emerged as a crucial component in promoting sustainable food systems. They serve as a sustainable and environmentally friendly substitute for animal-based protein sources. Meat production through livestock farming demands substantial quantities of resources, such as land, water, and feed, and is associated with deforestation and greenhouse gas emissions. In contrast, plant-based protein sources have a lower environmental impact, requiring fewer resources and generating fewer emissions [1,2]. In addition, plant-based proteins are instrumental in supporting human health by offering several advantages. They generally contain lower levels of saturated fats and cholesterol in comparison to animal-based proteins, thus contributing to cardiovascular well-being. Furthermore, plant-based proteins are often abundant in dietary fiber, vitamins, minerals, and phytochemicals, which provide a wide range of health benefits. These benefits include reducing the likelihood of chronic conditions like obesity, diabetes, and specific types of cancer [3,4].

On the other hand, pseudocereal grains such as quinoa (*Chenopodium quinoa*) are suitable crops and can contribute to sustainable food production. Quinoa belongs to the Amaranthaceae family and has been used as a primary ingredient in the diet of the ancient Andean region [5,6,7,8]. In recent years, quinoa seeds have gained recognition as a ‘superfood’ due to their exceptional nutritional and biological value. Quinoa seeds are highly valued for their superior protein content, comparable to certain cereal grains. Quinoa protein is particularly beneficial, as it is gluten-free, setting it apart from other cereal grains [9,10]. Quinoa is an exceptional source of nutrients, including dietary fiber, vitamins (e.g., vitamin E and B complex), minerals (e.g., magnesium, iron, and zinc), and antioxidants.

Additionally, this gluten-free grain boasts a low glycemic index, providing an excellent choice for both individuals with gluten sensitivities and those seeking to manage their blood sugar levels. With its versatile and nutrient-dense profile, quinoa is valuable to any healthy and well-balanced diet [10,11,12]. However, grains are generally considered to have lower protein content compared to some other protein-rich sources, such as legumes, meats, and certain dairy products [13]. While grains such as quinoa seeds do contain slightly higher protein content than other traditional grains [12], they are still not as concentrated or complete as the proteins found in animal-based foods or certain plant-based sources.

In this regard, food processing techniques, such as fermentation, soaking, and germination, have demonstrated a notable influence on the chemical composition of plants. Several studies have reported changes in protein quality following the application of fermentation or germination methods [14,15]. The germination process involves wetting the grain seeds, leading to increased hydration and affecting the structure and functionality of the seeds’ nutrients. Such effects can result in several biochemical changes, leading to major modifications in the nutritional profile of sprouted seeds [16]. Studies have shown that the germination process has the ability to promote the activation of multiple hydrolytic enzymes, leading to the breakdown of complex carbohydrates and proteins into simpler forms that are more easily digestible. The stimulated enzymatic activity might consequently contribute to an increase in the content of amino acids, thereby enhancing the overall protein quality of the sprouted seeds. Overall, the germination or sprouting process can be considered a valuable technique to optimize the nutritional benefits of grain seeds [14,17,18]. Therefore, this study aimed to examine the potential impact of the germination process on the protein quality of yellow and red quinoa seeds. The study addresses the potential of meeting the growing global demand for protein, highlighting plant-based proteins as a scalable and efficient solution.

## 2. Results

### 2.1. Germination Parameters

The germination parameters of RQ and YQ, including germination percentage, average time, and germination index, are presented in Table 1. After 6 days of germination at a temperature of 17 °C and a relative humidity (RH) of 90–93%, significant differences were noted between the two quinoa sprouts (*p* < 0.05). The most favorable germination parameters were recorded in RQ compared to YQ. The latter recorded a lower germination percentage of 25% compared to RQ (43.33 ± 1.53% vs. 57.76 ± 1.53%, respectively). The germination index of RQ was also higher than that of YQ by 17%. In addition, RQ required less average time for a sufficient germination process than YQ, as shown in Table 1.

**Figure 1:**
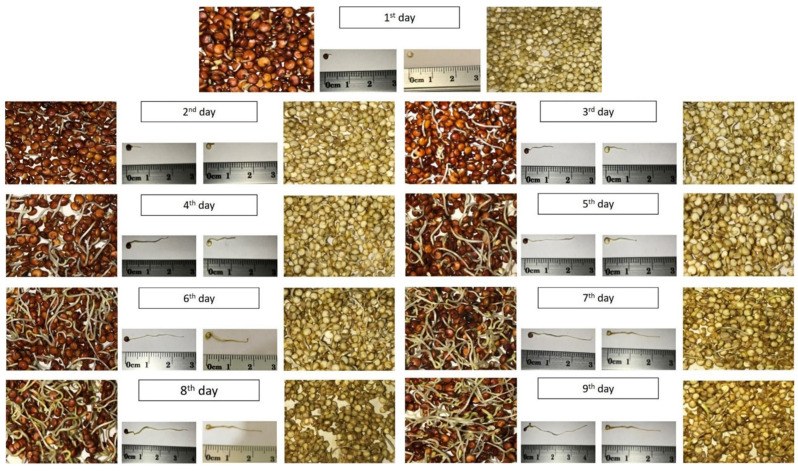
The stages of germination and plumula radicals’ development of YQ and RQ at 17 ± 1 °C and 90–93% RH for 9 days. YQ, the yellow-colored sprouts on the right side; and RQ, the red-colored sprouts on the left side.

Furthermore, the physical development stages of the two sprouted quinoa seeds were monitored during the sprouting process, as shown in Figure 1. The sprouting process of the RQ showed a higher growth rate than that observed with the YQ. After the third day of germination, the average length of plumula radicals of the RQ reached nearly 12 mm, while that of YQ reached only 9 mm. With the progression of germination time, the plumula radicals’ lengths of the RQ and YQ reached 25 mm and 18 mm after six days of germination. The growth development of the plumula radicals continued up to day 9 of germination, reaching a length of 41 and 24 mm for the RQ and YQ, respectively.

### 2.2. Content of Total Saponins during Germination

**Figure 2:**
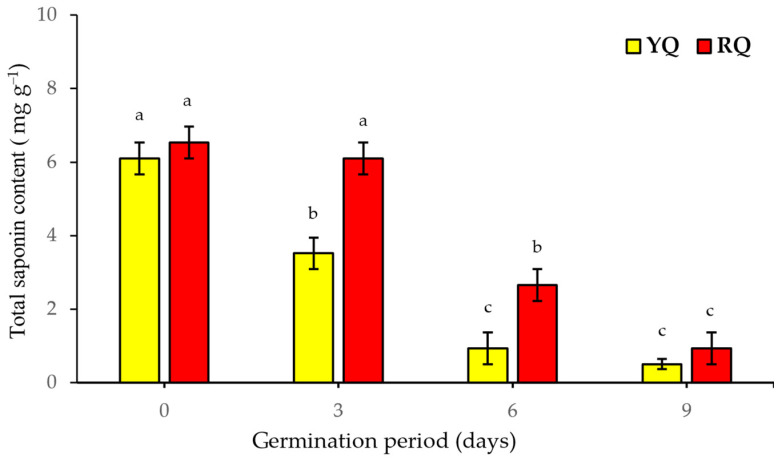
Total saponins content in YQ and RQ sprouts during germination for 9 days (17 ± 1 °C and 90–93% RH). ^a,b,c^ Bars not sharing similar letters are significantly different (*p* > 0.05).

The changes in total saponins (TS) during 9 days of germination were monitored, as shown in Figure 2. The results showed significant differences (*p* < 0.05) between the raw YQ seeds (day 0) and the sprouted YQ seeds after the third and sixth days of germination. The mean TS content showed a reduction of 42.2% and 84.7% after the germination process after 3 and 6 days, respectively. The TS continued to decrease until day 9 by 91.8%; however, this decrease was not statistically different from that observed on the sixth day, indicating that 6 days of germination might be optimal for reducing the content of TS. However, the RQ showed a significant (*p* < 0.05) reduction in the TS content from the sixth day up until the ninth day of germination compared to that recorded in the raw seeds (day 0). A reduction in TS by 6.58%, 59.2%, and 85.7% was obtained in the RQ after 3, 6, and 9 days of germination, respectively.

### 2.3. Content of α-Amylase and Protease during Germination

**Figure 3:**
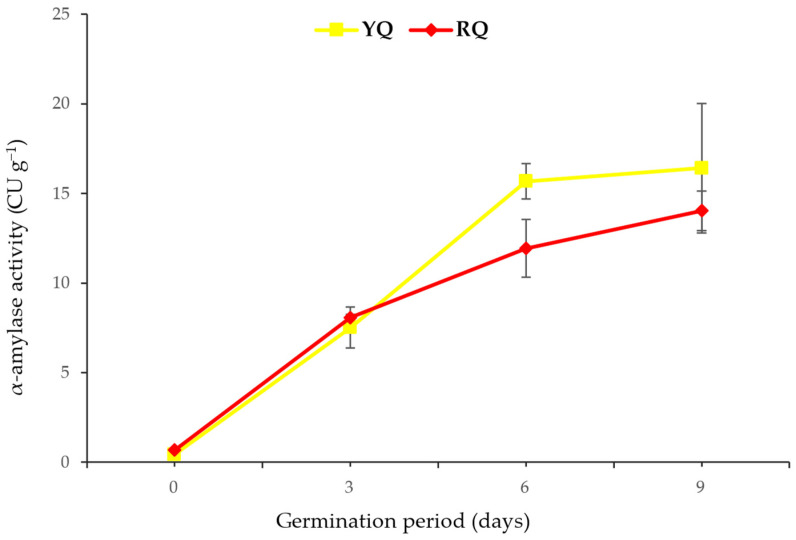
The activity of α-amylase in YQ and RQ sprouts during germination for 9 days (17 ± 1 °C and 90–93% RH). CU, Ceralpha Unit.

**Figure 4:**
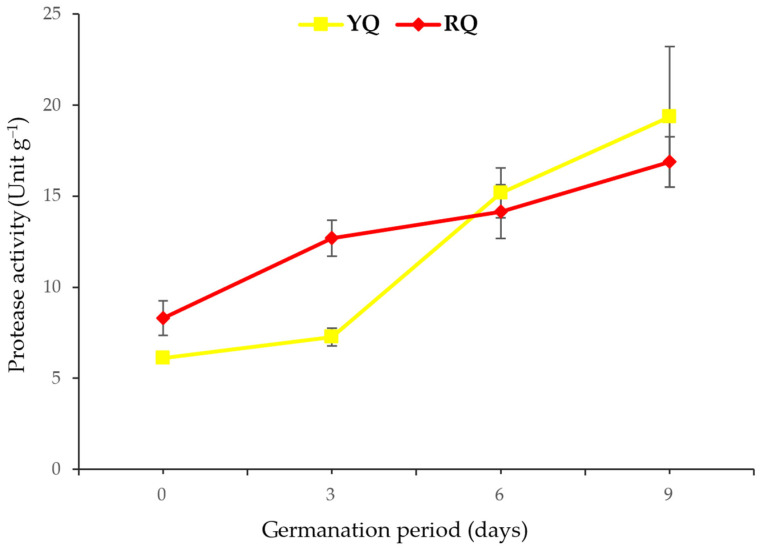
Protease enzyme activity in YQ and RQ sprouts during germination for 9 days (17 ± 1 °C and 90–93% RH).

The activity of α-amylase and protease enzymes of the two quinoa seeds and their sprouts was screened during a germination period of 9 days, as shown in Figure 3 and Figure 4. Figure 3 shows that there were significant changes (*p* < 0.05) in the activity of α-amylase recorded in the YQ starting from the third day of germination compared to the raw seeds (day 0). A significant increase (*p* < 0.05) in the α-amylase activity was consistent with the progression of germination time up to the sixth day. However, the further progression in the germination time up to day 9 showed nonsignificant changes (*p* > 0.05) in the α-amylase activity. It was indicated that 6 days of germination is optimal for improving the activity of α-amylase. Similar results were recorded in the RQ raw seeds and sprouts; the significant increase (*p* < 0.05) in the activity of α-amylase started from day 6 up to day 3 compared to that recorded in the raw seeds (day 0).

Further, the comparison of the overall mean content of α-amylase between the two quinoa sprouts indicated no significant differences (*p* > 0.05), regardless of the duration of germination. It is worth noting that a slight difference was observed on day 6. However, this difference had diminished by the end of the germination period and was no longer significant (*p* > 0.05). On the other hand, the protease enzyme activity also showed a significant increase (*p* < 0.05) in the RQ sprouts starting from day 3, and in the YQ, starting from day 6 (Figure 4). The two quinoa sprouts showed a consistent increase in the protease activity up to day 9 of germination. However, a significant difference (*p* < 0.05) was found in the overall mean content of protease between the two quinoa sprouts, regardless of the germination duration. Notably, both raw seeds exhibited similar protease content, indicating that germination resulted in a higher protease content in the YQ sprouts than in the RQ sprouts.

### 2.4. Composition of Amino Acids during Germination

The changes in the content of essential and non-essential amino acids in the raw seeds of the YQ and RQ and their sprouts were screened during the germination period, as presented in Table 2. The results showed that most of the essential amino acids screened in YQ sprouts recorded an increase after the third and sixth day of germination compared to raw, except for the histidine and methionine, which increased after day 3, with no further increase after day 6. Similar results were recorded in the RQ sprouts; most of the essential amino acids showed an increase after the third and sixth day of germination compared to raw, except the essential amino acid valine, which, on the contrary, showed a decline. The content of non-essential amino acids in YQ and RQ sprouts, including glutamic acid, aspartic, and proline, recorded a constant increase after the third and sixth day of germination compared to raw seeds. In comparison, the amino acid tyrosine showed a decline in the YQ sprouts after day 3 of germination but rose after the sixth day. Similarly, the amino acid alanine–tyrosine slightly decreased in the RQ sprouts after day 3 of germination but recorded a rise after day 6.

### 2.5. Quality of Protein during Germination

The quality of proteins in the two sprouted quinoa seeds was screened during the germination period, as presented in Table 3. The results were also compared to some highly processed animal proteins, such as beef and egg proteins (FAO, 1970) [19]. During the germination process, the percentage of essential amino acids showed an increase in a linear relationship with the increase in germination time compared to the raw seeds. This increase was higher in the RQ than in the YQ; an increase of 4.07 and 5.86% was recorded in the YQ after days 3 and 6, respectively, while the RQ recorded an increase of 5.97 and 15.67%, respectively. When comparatively analyzing the obtained results with the standard values estimated for beef and eggs, we found that the percentages of essential amino acids found in the YQ and RQ after 6 days of germination were slightly lower but closely resembled those of beef and eggs. In other words, the essential amino acids increased after germination, nearly approaching the levels found in beef and eggs. The percentage of the non-essential amino acids also showed an increase in both sprouted quinoa seeds after germination, as shown in Table 3. Similar to that observed with the essential amino acids, the non-essential amino acids also showed a higher increase in the RQ than in the YQ. The YQ recorded an increase of 4.66 and 8.68% after days 3 and 6, respectively, while the RQ recorded an increase of 7.60 and 13.25%, respectively.

Furthermore, the ratio between essential and non-essential amino acids (EAAs: NEAAs) showed a decrease in a linear relationship with the germination time. Both sprouted seeds recorded a decrease compared to the raw seeds. However, on the sixth day, the RQ exhibited a slight increase of 2.14%, surpassing the levels observed in the raw seeds. Comparatively analyzing the EAAs: NEAAs ratio of both sprouted seeds to that estimated in eggs and beef, we can see that the obtained results are less than that found in eggs but slightly reaching that found in beef. Moreover, Table 3 also shows that the ratio between essential amino acids and protein in the two sprouted seeds increased after 6 days of germination by 5.97% and 15.6% in YQ and RQ, respectively. However, these values are lower than those reported in eggs and beef.

On the other hand, the ratio between essential amino acids and total amino acids (EAAs: Total AA) showed a slight decrease in the two sprouted seeds. However, RQ showed a rise on the sixth day. The values of EAAs: Total AA are slightly less but nearly reaching that found in beef and eggs. Furthermore, the essential amino acids index showed an increase along with the germination time of the two sprouted seeds. When comparing the obtained results with beef and eggs, both sprouted seeds demonstrated lower indices than eggs but were similar to beef, with YQ showing an index almost identical to that of beef.

The ratio of each essential amino acid to the total essential amino acids was further estimated in the two quinoa seeds during germination time, as presented in Table 4. The results showed that the ratio of threonine, isoleucine, leucine, phenylalanine, valine, and lysine increased in YQ with the progression of germination time. Specifically, after the sixth day, the ratios of these amino acids increased by 15.04%, 14.4%, 13.78%, 13.09%, 10.15%, and 4.30%, respectively. Furthermore, the comparative analysis of specific amino acids, such as leucine, lysine, methionine, phenylalanine, threonine, and histidine, in YQ revealed that their ratios were higher than the standard ratios found in chicken eggs (Table 4). The RQ sprouts also exhibited consistent results, showing an increase in all essential amino acids with the progression of germination time. Cystine, leucine, histidine, phenylalanine, isoleucine, methionine, lysine, and threonine recorded an increase of 100.7%, 21.19%, 18.78%, 16.02%, 15.17%, 12.03%, 10.9%, and 9.41%, respectively. The comparative analysis of leucine, lysine, phenylalanine, histidine, and cysteine in the RQ sprouts revealed that their ratios were higher than those found in chicken eggs.

Furthermore, the results revealed some specific essential amino acids limiting the quality of protein, calculated as a percentage of the number of milligrams of each essential amino acid present in a gram of the protein content of the RQ and YQ sprouts (Table 5). There were three limiting amino acids with values less than 100; the first represented the combined value of cysteine and methionine, followed by threonine and phenylalanine. The three identified amino acids were observed in both YQ and RQ sprouts after day 3 and day 6 of germination. However, threonine was identified as a limiting amino acid only on the third day, not the sixth day.

## 3. Discussion

Plant-based proteins have emerged as vital protein sources due to their nutritional value, health benefits, environmental sustainability, ethical considerations, variety, and accessibility, as well as their potential to contribute to global food security. Sprouting has been demonstrated as a valuable food technique for enhancing the quality of plant proteins. The current study investigated the qualitative and quantitative changes in the amino acids of yellow and red quinoa seeds in addition to changes that occurred in some proteolytic enzymes.

Interestingly, the results showed that the sprouting process significantly (*p* < 0.05) increased total amino acids, including both essential and non-essential (EAA and NEAA), in the two quinoa varieties. After 6 days of sprouting, the total amino acids increased by 7.43% and 14.36% in the YQ and RQ, respectively. The observed new values are remarkably close to the standard protein content found in chicken eggs, as determined by the FAO [19]. The total NEAA content was higher in both sprouted YQ and RQ than in chicken eggs. However, both still contain fewer EAAs than chicken eggs, suggesting that chicken eggs remain a distinct source of high EAA content. Nevertheless, the significant increase (*p* < 0.05) observed in total amino acids indicates the efficiency of the sprouting process in improving the protein content of quinoa seeds. Similarly, the sprouting process of wheat and brown rice showed an increase in total EAA [20]. Other studies showed that sprouted wheat recorded an increase in both EAA and NEAA compared to raw data [21,22]. The sprouting of chia seeds effectively showed a consistent increase in both EAA and NEAA [23]. The sprouting process of the two quinoa seeds also showed an increase in the ratio of total EAA to the protein content. The results recorded a time-dependent manner, indicating that extending the sprouting duration up to six days can effectively enhance the protein quality of quinoa seeds. On the other hand, the ratio of EAA to total amino acids showed a decrease in the two sprouted seeds during the first stage of sprouting (day 3), followed by a significant increase after 6 days (*p* < 0.05), indicating the robust time-dependent trend. This increase was observed in the RQ seeds but not the YQ. However, it is worth noting that higher levels of NEAA were observed in the YQ throughout the sprouting process, exceeding those observed in the RQ. Therefore, the decrease in the ratio of EAA to total amino acids observed in the YQ resulted from increased levels of NEAA exceeding the EAA as the sprouting process progressed. Interestingly, these ratios almost approached the standard ratios found in beef and chicken eggs, with a difference ranging from 1.6% to 9% [19]. Therefore, sprouted quinoa seeds might serve as a high-quality plant-based protein source to replace animal proteins in vegetarian diets. These findings also indicate the potential possibility of a sprouting process for a wide range of grain seeds, as grains are generally recognized for their low content of EAAs, particularly tryptophan, lysine, and methionine [24,25]. An increase in the levels of lysine, as well as other EAAs (e.g., leucine, phenylalanine, threonine, and valine), was observed in the sprouted quinoa seeds. Some of the new values were higher than those found in the standard protein of chicken eggs, specifically leucine, phenylalanine, and histidine. A similar increase in the levels of isoleucine, leucine, phenylalanine, and valine was reported in waxy wheat after 1.5 days of sprouting, while other EAAs, such as threonine and methionine, reached higher levels after 3 days of sprouting [26]. The sprouting of rice and buckwheat malts for 4–5 days showed remarkable increases in multiple amino acids, both of which are known for their low content of amino acids, such as methionine and histidine [27]. In addition, the sprouting of oat grains was reported to promote an increase in the content of albumin (high in EAAs such as lysine and tryptophane) with a subsequent decrease in globulin and prolamin content (low in lysine) [28]. Essential amino acids such as threonine, methionine, and tryptophan play crucial roles in human health. Threonine, for instance, is a vital amino acid involved in numerous important bodily functions. It plays a vital role in processes like protein synthesis and metabolism. Threonine is an integral part of essential proteins like elastin and collagen, which contribute to the structural integrity of tissues such as skin, tendons, and cartilage [29]. Meanwhile, tryptophan serves as a precursor to serotonin, a neurotransmitter responsible for regulating mood, sleep, and appetite. It is also involved in the synthesis of melatonin, a hormone that regulates the sleep–wake cycle. Consequently, tryptophan plays a vital role in maintaining the proper functioning of the nervous system and has been associated with promoting relaxation, reducing anxiety, and enhancing overall mood [30,31]. Other amino acids like lysine have been well-demonstrated to contribute to various health-promoting properties. Lysine enhances immune function by aiding in the formation of antibodies, improving stomach function, facilitating cell repair, participating in fatty acid metabolism, and enhancing calcium absorption [26,32,33,34,35]. In the current study, the bioavailability of lysine, which is typically limited in grains, showed a notable increase in the two quinoa seeds after the sprouting process. The new levels exceeded those found in typical grains, such as wheat, rice, oats, and millet. Similarly, previous studies have reported a significant increase in the lysine content of brown rice and wheat during the sprouting process, with an observed increase of 33.90% and 10.05%, respectively [20].

On the other hand, it should be noted that certain EAAs have been identified as limiting amino acids that affect the protein quality of sprouted quinoa seeds. Three limiting amino acids were observed: the first is a combination of cysteine and methionine, followed by threonine and then phenylalanine. These results differ from those reported by Ruales and Nair [36]; they found that the first limiting amino acids that restrict quinoa seeds were the aromatic amino acids tyrosine and phenylalanine, followed by threonine and then lysine. However, they reported that the amounts of lysine and sulfur amino acids (cysteine + methionine) were relatively high. The observed improvements in the amino acid profile might be attributed to multiple possible mechanisms of action. The complete formation of amino acids during the sprouting process is a viable theory. However, the observed increase in amino acids is closely related to protein degradation due to activated protease enzymes as a result of increased seed hydration, leading to higher amino acid bioavailability [37]. Indeed, there are some minor challenges of this method; for instance, the high fat sample should be extracted, and accurate protein content should also be determined prior to the amino acid analysis. This method cannot determine the tryptophane, and different nutritional calculated results in Table 3, Table 4 and Table 5 depend on which amino acid profile data are used for comparison [38].

Increased seed hydration or water uptake during sprouting can effectively promote the activation of hydrolytic enzymes such as protease. Proteases, also known as peptidases or proteinases, are enzymes responsible for protein breakdown by cleaving the peptide bonds between amino acids [39]. These enzymes are normally found in an inactive state within the seed and require water to initiate their activity since water absorption during the germination process initiates a series of biochemical changes, leading to their activation [40,41]. Supporting these facts, a remarkable increase in the activity of protease was observed throughout the sprouting process of the two quinoa seeds in the current work. Initially, the raw quinoa seeds recorded 8.30 and 6.11 units g^−1^ of protease activity in the RQ and YQ, respectively. These results are less than that reported by Makinen et al. [42], who found protease activity of 9.6 units g^−1^ in raw quinoa seeds. In comparison, Lorenz and Nyanzi [43] reported similar protease activity of 5.5–8.9 μg of tyrosine g^−1^ in the raw quinoa seeds. These variations in protease activity could be attributed to different factors, such as quinoa variety, growing conditions, sample preparation, and assay methods used in different studies. The sprouting process significantly increased the protease activity (*p* < 0.05), with RQ exhibiting a higher content than that of YQ. However, on the sixth day of germination, a notable and significant spike (*p* < 0.05) in protease activity was observed in the YQ, exceeding that found in the RQ, indicating a distinct enzymatic response during this particular stage of germination. Higher protease activity has also been reported in various sprouted grains, including oats, barley, rye, and wheat. The activity increases progressively with the duration of sprouting, indicating a time-dependent rise in protease activity [44]. Similarly, other enzymes, like amylase, have been found to increase during grain sprouting. In sprouted corn, α-amylase activity was reported to be 2.87 times higher after 7 days of germination compared to 1 day [45]. In the current study, the sprouting process of up to 9 days resulted in an increase in the activity of α-amylase in both sprouted quinoa seeds. However, the increase observed on the ninth day was not significantly different (*p* > 0.05) from that observed on the sixth day of germination, suggesting that six days of sprouting may be sufficient to promote enzymatic activity. In a previous study, 3 days of sprouting quinoa seeds showed a remarkable increase in the α-amylase activity [46]. Other multiple studies showed a similar increase in the α-amylase activity from 0 to 3.5 units within 3 days of germination [42,47]. Interestingly, higher α-amylase content may contribute to increased stickiness and cohesiveness in the seeds [48,49]. Indeed, it was observed that YQ exhibited greater stickiness compared to RQ, supporting the correlation between α-amylase content and sprouted seed texture.

The observed enzymatic activity can be affected by multiple factors other than seed hydration; factors such as temperature can also play a role in influencing enzymatic activation during the sprouting process. Optimal temperatures have a direct impact on the overall growth and development of the plant, therefore influencing the degree of the bioactivity of the chemical reactions. Rosa et al. [50] reported that proceeding with the sprouting process at a temperature of approximately 25 °C showed significantly higher enzymatic activity, while at 5 °C, no noticeable changes in enzyme activity were observed. In the current work, the sprouting process was carried out at 17 ± 1 °C. At this temperature, the growth rate of both seeds successfully showed higher rates of nearly 58% and 43% in the RQ and YQ, respectively. A compatible germination rate in the range of 45–50% was reported for quinoa seeds sprouted at 22 °C in a pilot study by Schlick and Bubenheim [51]. Another study reported a remarkably higher maximum germination rate, reaching 87% at a temperature of 15 °C [47]. In the current work, a notable disparity in the germination progression between the RQ and YQ varieties was observed; the RQ exhibited a faster germination rate of 14% higher than that of the YQ. This disparity may account for the observed increase in all EAAs recorded in the RQ variety, while no increase was observed in the YQ variety. The observed variations in seed germination rates can be related to genetic variability, as well as cultivation conditions. The cultivation conditions encompass various environmental factors, such as light, temperature, moisture, and soil quality, which directly impact the germination process [52]. To develop a comprehensive understanding of quinoa seed germination processes, future studies should consider both genetic variability and cultivation conditions.

From another point of view, increased enzymatic activity during germination might also relate to a reduction in some inhibitory substances, such as saponins. Saponins are natural secondary compounds derived from various plants and exhibit various biological activities. Saponins have also been reported to promote inhibitory activities on various digestive enzymes, including amylase, glucosidase, and proteases such as trypsin and chymotrypsin. These saponins can bind to the active sites of these enzymes, thus preventing substrate binding and inhibiting their catalytic activity [39,53]. Saponins are also associated with a bitter taste, which may limit the use of quinoa. Processes such as washing and scrubbing to remove the bitter taste are effectively correlated with a reduction in saponin content of 60% to almost unreachable amounts [54]. The reduction of saponins can also be achieved through other processes, including soaking, thermal extrusion, roasting, or germination [55]. Lorenz and Nyanzi [43] demonstrated that, by removing the quinoa’s pericarp (outer layers), which is the main source of saponins, through a combination of soaking and heat treatments, the protease activity of quinoa increased due to the reduction in saponin content. In the current study, the saponin content of the two sprouted quinoa seeds showed similar results. The increase in the seeds’ hydration due to germination led to a significant decrease (*p* < 0.05) in the saponins’ content in a time-dependent manner. After six-to-nine days of germination, a reduction of nearly 85–92% and 59–86% in total saponin content was observed in the YQ and RQ, respectively. These results support the fact that germination facilitates water penetration into the seeds, resulting in the release of saponins through simple diffusion [56]. A study conducted on black beans revealed that the concentration of saponins decreased as the germination period increased. It was found that one day of germination was sufficient to enhance the concentration of saponins [57]. The sprouting process can also result in the degradation of other anti-nutrients, such as phytic acid; it was reported that sprouted quinoa seeds showed a decrease of 32–74% in phytic acid content [58]. Therefore, the germination process holds promising potential as a valuable technique for enhancing the nutritional value of quinoa seeds.

## 4. Materials and Methods

### 4.1. Ingredients

Two varieties of quinoa (*Chenopodium quinoa*), yellow (YQ) and red (RQ), were obtained from the Al-Tamimi market located in Qassim, Saudi Arabia. The YQ was produced in Peru and exported by Bode Naturkost Co. (Hamburg, Germany), while the RQ was produced in the USA and exported by Now Foods Inc. (Bloomingdale, IL, USA).

### 4.2. Sprouting Process of Quinoa Seeds

The sprouting process was carried out following the method described by Al Qabba et al. [59]. Prior to the sprouting process, any dust and broken seeds were carefully eliminated, and the seeds underwent thorough cleaning. Following the cleaning procedure, the seeds were sprouted immediately and collected at three specific time points: 3, 6, and 9 days. The sprouting process was carried out under a controlled temperature of 17 ± 1 °C and relative humidity (RH) of 90–93%, using the sprouting machine. The sprouts were frozen overnight at 18 ± 1 °C and then subjected to freeze-drying for 96 h, at −48 °C and 0.032 mbar (CHRIST, Alpha 1–2 LD plus, Osterode, Germany). A fine powder was prepared by grinding freeze-dried sprouts in a small mill (Thomas Wiley, Philadelphia, PA, USA) and sieving the resulting mixture through a 60-mesh sieve. The powder was then stored in dark containers at 4 ± 1 °C, until it was used for chemical analysis. To biologically evaluate quinoa sprouts, 2 kg of RQ and YQ sprouts was collected after 6 days; progressively dried for 24 h, according to Barakat et al. [60]; pulverized; sieved; and stored under cooling until the extraction.

### 4.3. Determination of Total Saponins (TS)

The TS concentration was determined using the Koziol afrosimetric technique, as described by Morillo et al. [61]. In a 15 cm long, 15 mm diameter test tube, 0.5 g of seeds and 5 mL of distilled water were added. The tube was then covered briskly for 30 s; rested for 30 min; stirred again for 20 s; rested for 30 min; stirred again for 30 s, with intense agitation; and rested for another 5 min. Foam height was measured with a 0.1 cm ruler, and TS was reported as mg g^−1^ quinoa on dry base weight (mg g^−1^ DW), using the relevant equations.

### 4.4. Determination of α-Amylase and Protease Enzyme

#### 4.4.1. Extraction of α-Amylase

The extraction process described by McCleary et al. [62] was followed. A total of 0.5 g of quinoa malt was mixed with a solution of NaCl (1%, *w*/*v*) plus CaCl_2_ × 2H_2_O (0.03%, *w*/*v*) and left for 10 min at room temperature, with occasional swirling, and then centrifuged at 1000× *g* for 10 min. Clear supernatant was collected and diluted with sodium malate buffer (pH 5.4). After that, a substrate solution was preincubated at 40 °C for 5 min. Both buffered and diluted quinoa malt extracts were preincubated at 40 °C for 5 min. Place preequilibrated quinoa malt extract at the bottom of the substrate solution tube. Mix tube contents thoroughly with a vortex mixer and incubate at 40 °C for 10 min after addition. A stopped reagent was added after incubation, and tube contents were vigorously mixed. At 400 nm, solution and reaction blank absorbance vs. water were measured.

#### 4.4.2. Calculations of α-Amylase

The calculation of α-amylase was described by McCleary et al. [62], whereas the α-amylase content as Ceralpha Unit per g (CU g^−1^) was calculated using the following:

(1) $$\alpha \text{-}Amylase(\mathrm{Ceralpha}\mathrm{Unit}g^{-1})=\frac{A_{a}-A_{b}}{I}\times \frac{V_{r}}{Z}\times \frac{1}{\varepsilon }\times \frac{V_{e}}{W}\times F$$

where A_a_ = average absorbance of the two assay solutions at 400 nm; A_b_ = absorbance of reaction blank at 400 nm; I = 10 min; V_r_ = 3.4 mL; Z = 0.2 mL; e = the absorptivity of *p*-nitrophenol at 400 nm in 2% trisodium phosphate solution, pH 11.0; absorbance values are measured in a 10 mm spectrophotometer cuvette; V_e_ = 100 mL; W = 0.5 g; and F = extraction solution dilution factor.

#### 4.4.3. Extraction of Protease Enzyme

For extraction, 0.5 g of quinoa malt was combined with 9 mL of potassium phosphate buffer (pH 7.5), refrigerated for 30 min, and then centrifuged at 1000× *g* for 10 min. To measure enzyme activity, the supernatant was collected for the determination.

#### 4.4.4. Calculations of Protease Enzyme

The calculation of protease enzyme is described by Cupp-Enyard [63]. For 10 min at 37 °C, 5 mL of a casein solution at 0.65% (*w*/*v*) was combined with 1 mL of extracted protease. The reaction was terminated by adding 5 mL of a Trichloroacetic acid solution reagent (110 mM). The enzyme solution was incubated at 37 °C for 30 min, 0.45 µm polyethersulfone filtered, mixed with sodium carbonate and Folin’s reagent immediately as (2:5:1), and then incubated for 30 min at 37 °C. Finally, the absorbance of the filtered mixture was measured at 660 nm. The enzyme activity in units per mL was calculated using the relevant equation.

### 4.5. Determination of Amino Acids

The amino acid content of quinoa sprouts was analyzed by an amino acid analyzer (Sykam S 433 Amino Acid Analyzer, Sykam GmbH, Eresing, Germany), as described by Le et al. [64], with cation-exchange chromatography–ninhydrin postcolumn derivatization after submitting samples to acid hydrolysis (HCl 6 mol L^−1^, 110 °C, 24 h) [26].

### 4.6. Statistical Analysis

For the statistical analysis, SPSS 22.0 for Windows was used. Mean ± SE was the statistical measure used to summarize the outcomes of the experiments. Statistical significance was determined using a one-way analysis of variance (ANOVA) and a post hoc test, with *p*-values < 0.05 being considered to be significant, following Steel [65].

## 5. Conclusions

The current study revealed that the sprouting process can effectively improve the protein quality of yellow and red quinoa seeds. An increase of 7–14% in the total amino acids was observed after 6 days of sprouting. The content of various essential amino acids, including tryptophan, methionine, and lysine, has remarkably increased. The germination process was effective in increasing protease activity, leading to the observed results. Additionally, anti-nutrient components such as saponins showed a remarkable decline during the germination process. This study demonstrates that sprouted quinoa seeds might serve as an excellent plant-based protein source in vegetarian diets. Future studies should prioritize conducting additional investigations to establish a sustainable source of sprouted quinoa seeds and explore optimal storage methods, as well as the effects of storage on their nutritional quality. Expanding research efforts to examine the impact of genetic variations on the obtained results is also recommended.

## Figures and Tables

**Table 1:** Germination parameters of YQ and RQ after 6 days of germination at 17 °C ± 1 and 90–93% RH.

Item	Germination %	Mean Time of Germination	Germination Index
YQ	43.33 ± 1.53 ^b^	2.43 ± 0.19 ^a^	4.13 ± 0.32 ^b^
RQ	57.76 ± 1.53 ^a^	2.00 ± 0.02 ^b^	5.00 ± 0.05 ^a^

YQ, yellow quinoa; RQ, red quinoa. Results expressed as mean ± SE; different superscripted letters (^a,b^) in the same column are significantly different (*p* > 0.05).

**Table 2:** Amino acid composition of (g g^−1^ N) * in YQ and RQ seeds and sprouts during germination for 6 days at 17 ± 1 °C and 90–93% RH, compared to standard protein and amino acids scores.

Amino Acid		YQ				RQ				Eggs (FAO, 1970) [19]	
		0	3	6	SE	0	3	6	SE		
		Essential Amino Acids (EAAs)									
NAAs	Threonine	0.231 ^b^	0.257 ^a^	0.266 ^a^	0.011	0.220 ^c^	0.233 ^b^	0.240 ^a^	0.006	0.32	
NAAs	Cystine	0.182 ^a^	0.134 ^b^	0.125 ^b^	0.018	0.104 ^b^	0.133 ^b^	0.208 ^a^	0.031	0.152	
HAAs	Valine	0.309 ^b^	0.336 ^a^	0.340 ^a^	0.010	0.360 ^a^	0.329 ^b^	0.336 ^b^	0.009	0.428	
HAAs	Isoleucine	0.243 ^c^	0.257 ^b^	0.278 ^a^	0.010	0.232 ^c^	0.244 ^b^	0.267 ^a^	0.010	0.393	
HAAs	Leucine	0.413 ^b^	0.459 ^a^	0.470 ^a^	0.017	0.396 ^c^	0.430 ^b^	0.480 ^a^	0.024	0.551	
HAAs	Phenylalanine	0.276 ^b^	0.280 ^b^	0.312 ^a^	0.011	0.262 ^b^	0.271 ^b^	0.304 ^a^	0.013	0.358	
HAAs	Methionine	0.176 ^b^	0.179 ^a^	0.176 ^b^	0.001	0.152 ^b^	0.170 ^a^	0.171 ^a^	0.006		
BAAs	Lysine	0.380 ^b^	0.392 ^a^	0.397 ^a^	0.005	0.341 ^c^	0.366 ^b^	0.379 ^a^	0.011	0.436	
BAAs	Histidine	0.198 ^b^	0.213 ^a^	0.187 ^c^	0.008	0.171 ^b^	0.196 ^a^	0.203 ^a^	0.010	0.152	
		Non-Essential Amino Acids (NEAAs)									
AAAs	Aspartic acid	0.551 ^c^	0.565 ^b^	0.578 ^a^	0.008	0.530 ^b^	0.600 ^a^	0.624 ^a^	0.028	0.601	
AAAs	Glutamic acid	0.904 ^b^	1.024 ^a^	1.060 ^a^	0.047	0.835 ^b^	0.944 ^a^	0.987 ^a^	0.045	0.796	
NAAs	Serine	0.237 ^b^	0.291 ^a^	0.306 ^a^	0.021	0.244 ^b^	0.255 ^a^	0.235 ^c^	0.006	0.478	
NAAs	Tyrosine	0.231 ^a^	0.168 ^b^	0.238 ^a^	0.022	0.238 ^b^	0.228 ^b^	0.294 ^a^	0.021	0.26	
HAAs	Proline	0.237 ^b^	0.246 ^a^	0.249 ^a^	0.004	0.220 ^c^	0.239 ^b^	0.251 ^a^	0.009	0.26	
HAAs	Glycine	0.347 ^a^	0.347 ^a^	0.334 ^a^	0.004	0.317 ^c^	0.329 ^b^	0.336 ^a^	0.006	0.207	
HAAs	Alanine	0.309 ^c^	0.330 ^b^	0.368 ^a^	0.017	0.311 ^b^	0.302 ^b^	0.352 ^a^	0.015	0.37	
BAAs	Arginine	0.562 ^a^	0.565	0.538 ^b^	0.009	0.500 ^b^	0.541 ^a^	0.539 ^a^	0.013	0.381	
Total EAAs		2.409 ^c^	2.507 ^b^	2.550 ^a^	0.042	2.238 ^c^	2.372 ^b^	2.589 ^a^	0.102	3.218	
Total NEAAs		3.379 ^c^	3.536 ^b^	3.672 ^a^	0.085	3.195 ^c^	3.438 ^b^	3.619 ^a^	0.123	3.093	
Total amino acids		5.787 ^c^	6.043 ^b^	6.222 ^a^	0.126	5.433 ^c^	5.810 ^b^	6.207 ^a^	0.224	6.311	

* Calculated based on fresh weight. N, nitrogen; YQ, yellow quinoa; RQ, red quinoa; AAAs, acidic amino acids; BAAs, basic amino acids; HAAs, hydrophobic amino acids; NAAs, neutral amino acids; ES, standard error. ^a,b,c^ The values with the same superscripted letters in the same row inside each quinoa variety are not significantly different.

**Table 3:** Nutritional evaluation of essential and non-essential amino acids in YQ and RQ seeds and sprouts during germination for 6 days at 17 ± 1 °C and 90–93% RH.

Items	TEAA g 16 N^−1^	TNEAA g 16 N^−1^	EAAs: NEAAs	EAAs: Protein	EAAs: Total AA	EAAI %
YQ-0	38.54 ^c^	54.06 ^c^	0.71 ^a^	0.39 ^b^	0.42 ^a^	77.84 ^b^
YQ-3	40.11 ^b^	56.58 ^b^	0.71 ^a^	0.40 ^ab^	0.41 ^b^	77.85 ^b^
YQ-6	40.80 ^a^	58.75 ^a^	0.69 ^b^	0.41 ^a^	0.41 ^b^	79.03 ^a^
SE	0.67	1.36	0.01	0.1	0.00	0.40
RQ-0	35.80 ^c^	51.12 ^c^	0.70 ^b^	0.36 ^c^	0.41 ^b^	68.95 ^b^
RQ-3	37.95 ^b^	55.01 ^b^	0.69 ^b^	0.38 ^b^	0.41 ^b^	75.41 ^a^
RQ-6	41.42 ^a^	57.90 ^a^	0.72 ^a^	0.41 ^a^	0.42 ^a^	77.84 ^a^
SE	1.64	1.97	0.01	0.01	0.00	2.66
Egg (FAO, 1970) [19]	43.709	45.571	1.179	0.537	0.451	100.00
Beef (FAO, 1970) [19]	42.724	57.276	0.746	0.427	0.427	79.55

YQ, yellow quinoa; RQ, red quinoa; TEAA, total essential amino acids; TNEAA, total non-essential amino acid; EAAs: NEAAs, the ratio of essential amino acids to non-essential amino acid; EAAs: Protein Ratio; ratio of essential amino acids to 100 g protein; NEAAs: Total AA Ratio; ratio of non-essential amino acids to total amino acids; EAAI %: essential amino acids index according to FAO, 1970 [19]; ES, standard error. ^a,b,c^ The values with the same superscripted letters in the same column inside each quinoa variety are not significantly different.

**Table 4:** The ratio of individual essential amino acids to the total essential amino acids of YQ and RQ seeds and sprouts during germination for 6 days at 17 ± 1 °C and 90–93% RH, in comparison to reference EAA of egg protein (mg individual AA g^−1^ TEAA).

Amino Acid	YQ				RQ				Eggs (FAO, 1970) [19]
	0	3	6	SE	0	3	6	SE	
Threonine	96.11 ^c^	106.87 ^b^	110.57 ^a^	4.34	91.14 ^c^	96.93	99.72 ^a^	2.53	110.42
Valine	128.15 ^b^	139.39 ^a^	141.16 ^a^	4.08	149.37 ^a^	136.58 ^b^	139.61 ^b^	3.86	147.69
Isoleucine	100.69 ^c^	106.87 ^b^	115.28 ^a^	4.23	96.20 ^c^	101.33 ^b^	110.80 ^a^	4.28	135.61
Leucine	171.62 ^b^	190.50 ^a^	195.27 ^a^	7.23	164.56 ^c^	178.43 ^b^	199.44 ^a^	10.15	190.13
Phenylalanine	114.42 ^b^	116.16 ^b^	129.40 ^a^	4.74	108.86 ^b^	112.35 ^b^	126.31 ^a^	5.34	123.53
Lysine	157.89 ^c^	162.62 ^b^	164.69 ^a^	2.01	141.77 ^b^	152.00 ^a^	157.34 ^a^	4.57	150.45
Histidine	82.38 ^b^	88.28 ^a^	77.64 ^c^	3.08	70.89 ^b^	81.51 ^a^	84.21 ^a^	4.07	52.45
Cystine	75.51 ^a^	55.76 ^b^	51.76 ^b^	7.35	43.04 ^b^	55.07 ^b^	86.42 ^a^	12.95	52.45
Methionine	73.23 ^c^	74.34 ^a^	72.93 ^b^	0.43	63.29 ^b^	70.49 ^a^	70.91 ^a^	2.48	72.46

YQ, yellow quinoa; RQ, red quinoa; AA, amino acid; TEAA, total essential amino acids; ES, standard error. ^a,b,c^ The values with the same superscripted letters in the same row inside each quinoa variety are not significantly different. Amino acid score according to FAO (1970) [19] = $\frac{\mathrm{mg}\mathrm{amino}\mathrm{acid}\mathrm{in}1\;g\mathrm{protein}}{\mathrm{mg}\mathrm{amino}\mathrm{acid}\mathrm{suggested}\mathrm{by}\mathrm{FAO}/\mathrm{WHO}}\times 100.$

**Table 5:** Scores of essential amino acids in YQ and RQ seeds and sprouts during germination for 6 days at 17 ± 1 °C and 90–93% RH.

Amino Acid	YQ				RQ				Eggs (FAO, 1970) [19]
	0	3	6	SE	0	3	6	SE	
Threonine	92.59 ^b^	102.95 ^a^	106.53 ^a^	4.18	79.37 ^b^	97.00 ^a^	99.21 ^a^	6.28	40
Valine	98.77 ^b^	107.43 ^a^	108.79 ^a^	3.14	104.06 ^b^	109.35 ^a^	111.11 ^a^	2.12	50
Isoleucine	97.00 ^b^	102.95 ^b^	111.06 ^a^	4.08	83.77 ^c^	101.41 ^b^	110.23 ^a^	7.79	40
Leucine	94.48 ^b^	104.87 ^a^	107.50 ^a^	3.98	81.88 ^c^	102.04 ^b^	113.38 ^a^	9.22	70
Phenylalanine	91.86 ^b^	93.26 ^b^	103.88 ^a^	3.80	79.00 ^c^	93.69 ^b^	104.72 ^a^	7.46	35
Lysine	108.65 ^c^	111.91 ^b^	113.33 ^a^	1.39	88.18 ^b^	108.65 ^a^	111.80 ^a^	7.41	48
Histidine	151.17 ^b^	162.00 ^a^	142.47 ^c^	5.66	117.58 ^b^	155.37 ^a^	159.57 ^a^	13.37	55
Cystine	64.67 ^a^	47.75 ^b^	44.32 ^b^	6.30	33.31 ^c^	48.99 ^b^	76.43 ^a^	12.62	21
Methionine	62.71 ^b^	63.66 ^a^	62.46 ^b^	0.37	48.99 ^b^	62.71 ^a^	62.71 ^a^	4.58	-
Cystine + methionine	62.71 ^a^	62.71 ^a^	60.75 ^b^	0.65	48.99 ^b^	62.71 ^a^	62.71 ^a^	4.58	-

YQ, yellow quinoa; RQ, red quinoa; ES, standard error. ^a,b,c^ The values with the same superscripted letters in the same row inside each quinoa variety are not significantly different. Amino acid score according to FAO (1970) [19] = $\frac{\mathrm{mg}\mathrm{amino}\mathrm{acid}\mathrm{in}1\;g\mathrm{protein}}{\mathrm{mg}\mathrm{amino}\mathrm{acid}\mathrm{suggested}\mathrm{by}\mathrm{FAO}/\mathrm{WHO}}\times 100.$

## Data Availability

Data are contained within the article.

## References

[1] Langyan S., Yadava P., Khan F.N., Dar Z.A., Singh R., Kumar A. (2022). Sustaining Protein Nutrition through Plant-Based Foods. Front. Nutr..

[2] Hoehnel A., Zannini E., Arendt E.K. (2022). Targeted Formulation of Plant-Based Protein-Foods: Supporting the Food System’s Transformation in the Context of Human Health, Environmental Sustainability and Consumer Trends. Trends Food Sci..

[3] Ahnen R.T., Jonnalagadda S.S., Slavin J.L. (2019). Role of Plant Protein in Nutrition, Wellness, and Health. Nutr. Rev..

[4] Hertzler S.R., Lieblein-Boff J.C., Weiler M., Allgeier C. (2020). Plant Proteins: Assessing Their Nutritional Quality and Effects on Health and Physical Function. Nutrients.

[5] Angeli V., Miguel Silva P., Crispim Massuela D., Khan M.W., Hamar A., Khajehei F., Graeff-Hönninger S., Piatti C. (2020). Quinoa (*Chenopodium quinoa* Willd.): An Overview of the Potentials of the “Golden Grain” and Socio-Economic and Environmental Aspects of Its Cultivation and Marketization. Foods.

[6] Belton P.S., Taylor J.R. (2002). Pseudocereals and Less Common Cereals: Grain Properties and Utilization Potential.

[7] Rosentrater K.A., Evers A. (2018). Chapter 1—Introduction to Cereals and Pseudocereals and Their Production, In Kent’s Technology of Cereals.

[8] Vega-Gálvez A., Miranda M., Vergara J., Uribe E., Puente L., Martínez E.A. (2010). Nutrition Facts and Functional Potential of Quinoa (*Chenopodium quinoa* Willd.), an Ancient Andean Grain: A Review. J. Sci. Food Agric..

[9] Choque-Quispe D., Ligarda-Samanez C.A., Ramos-Pacheco B.S., Leguía-Damiano S., Calla-Florez M., Zamalloa-Puma L.M., Colque-Condeña L. (2021). Phenolic Compounds, Antioxidant Capacity, and Protein Content of Three Varieties of Germinated Quinoa (*Chenopodium quinoa* Willd). Ing. Investig..

[10] Pathan S., Siddiqui R.A. (2022). Nutritional Composition and Bioactive Components in Quinoa (*Chenopodium quinoa* Willd.) Greens: A Review. Nutrients.

[11] Navruz-Varli S., Sanlier N. (2016). Nutritional and health benefits of quinoa (*Chenopodium quinoa* Willd.). J. Cereal Sci..

[12] Fischer S., Wilckens R., Jara J., Aranda M., Valdivia W., Bustamante L., Graf F., Obal I. (2017). Protein and Antioxidant Composition of Quinoa (*Chenopodium quinoa* Willd.) Sprout from Seeds Submitted to Water Stress, Salinity and Light Conditions. Ind. Crops Prod..

[13] Poutanen K.S., Kårlund A.O., Gómez-Gallego C., Johansson D.P., Scheers N.M., Marklinder I.M., Eriksen A.K., Silventoinen P.C., Nordlund E., Sozer N. (2022). Grains–a Major Source of Sustainable Protein for Health. Nut. Rev..

[14] Nkhata S.G., Ayua E., Kamau E.H., Shingiro J.B. (2018). Fermentation and Germination Improve Nutritional Value of Cereals and Legumes through Activation of Endogenous Enzymes. Food Sci. Nutr..

[15] Bera I., O’Sullivan M., Flynn D., Shields D.C. (2023). Relationship between Protein Digestibility and the Proteolysis of Legume Proteins During Seed Germination. Molecules.

[16] Benincasa P., Falcinelli B., Lutts S., Stagnari F., Galieni A. (2019). Sprouted Grains: A Comprehensive Review. Nutrients.

[17] Ikram A., Saeed F., Afzaal M., Imran A., Niaz B., Tufail T., Hussain M., Anjum F.M. (2021). Nutritional and End-Use Perspectives of Sprouted Grains: A Comprehensive Review. Food Sci. Nutr..

[18] Lemmens E., Moroni A.V., Pagand J., Heirbaut P., Ritala A., Karlen Y., Lê K.A., Van den Broeck H.C., Brouns F.J., De Brier N. (2019). Impact of Cereal Seed Sprouting on Its Nutritional and Technological Properties: A Critical Review. Compr. Rev. Food Sci. Food Saf..

[19] Food and Agriculture Organization (FAO) (1970). Food and Agriculture Organization (FAO). Food Policy and Food Science Service. Amino-acid Content of Foods and Biological Data on Proteins. Amino-Acid Content of Foods and Biological Data on Proteins.

[20] Sibian M.S., Saxena D.C., Riar C.S. (2017). Effect of Germination on Chemical, Functional and Nutritional Characteristics of Wheat, Brown Rice and Triticale: A Comparative Study. J. Sci. Food Agric..

[21] Hung P.V., Maeda T., Yamamoto S., Morita N. (2012). Effects of Germination on Nutritional Composition of Waxy Wheat. J. Sci. Food Agric..

[22] Van Hung P., Maeda T., Morita N. (2015). Improvement of Nutritional Composition and Antioxidant Capacity of High-Amylose Wheat During Germination. J. Food Sci. Technol..

[23] Gomez-Favela M.A., Gutierrez-Dorado R., Cuevas-Rodriguez E.O., Canizalez-Roman V.A., Del Rosario Leon-Sicairos C., Milan-Carrillo J., Reyes-Moreno C. (2017). Improvement of Chia Seeds with Antioxidant Activity, Gaba, Essential Amino Acids, and Dietary Fiber by Controlled Germination Bioprocess. Plant Foods Hum. Nutr..

[24] Wenefrida I., Utomo H.S., Blanche S.B., Linscombe S.D. (2009). Enhancing Essential Amino Acids and Health Benefit Components in Grain Crops for Improved Nutritional Values. Recent Pat. DNA Gene Seq..

[25] Siddiqi R.A., Singh T.P., Rani M., Sogi D.S., Bhat M.A. (2020). Diversity in Grain, Flour, Amino Acid Composition, Protein Profiling, and Proportion of Total Flour Proteins of Different Wheat Cultivars of North India. Front. Nutr..

[26] Aggarwal R., Bains K. (2022). Protein, Lysine and Vitamin D: Critical Role in Muscle and Bone Health. Crit. Rev. Food Sci. Nutr..

[27] Agu R., Chiba Y., Goodfellow V., Mackinlay J., Brosnan J., Bringhurst T., Jack F., Harrison B., Pearson S., Bryce J. (2012). Effect of Germination Temperatures on Proteolysis of the Gluten-Free Grains Rice and Buckwheat During Malting and Mashing. J. Agric. Food Chem..

[28] Kaukovirta-Norja A., Wilhelmson A., Poutanen K. (2004). Germination: A Means to Improve the Functionality of Oat. Agric. Food Sci..

[29] Tang Q., Tan P., Ma N., Ma X. (2021). Physiological Functions of Threonine in Animals: Beyond Nutrition Metabolism. Nutrients.

[30] Kałużna-Czaplińska J., Gątarek P., Chirumbolo S., Chartrand M.S., Bjørklund G. (2019). How Important Is Tryptophan in Human Health?. Crit. Rev. Food Sci. Nutr..

[31] Höglund E., Øverli Ø., Winberg S. (2019). Tryptophan Metabolic Pathways and Brain Serotonergic Activity: A Comparative Review. Front. Endocrinol..

[32] Huang D., Maulu S., Ren M., Liang H., Ge X., Ji K., Yu H. (2021). Dietary Lysine Levels Improved Antioxidant Capacity and Immunity Via the Tor and P38 Mapk Signaling Pathways in Grass Carp, Ctenopharyngodon Idellus Fry. Front. Immunol..

[33] Lopez M.J., Mohiuddin S.S. (2023). Biochemistry, Essential Amino Acids.

[34] Unni U.S., Raj T., Sambashivaiah S., Kuriyan R., Uthappa S., Vaz M., Regan M.M., Kurpad A.V. (2012). The Effect of a Controlled 8-Week Metabolic Ward Based Lysine Supplementation on Muscle Function, Insulin Sensitivity and Leucine Kinetics in Young Men. Clin. Nutr..

[35] Hayamizu K., Oshima I., Nakano M. (2020). Comprehensive Safety Assessment of L-Lysine Supplementation from Clinical Studies: A Systematic Review. J. Nutr..

[36] Ruales J., Nair B.M. (1992). NAIR. Nutritional Quality of the Protein in Quinoa (*Chenopodium quinoa*, Willd) Seeds. Plant Foods Hum. Nutr..

[37] Kuo Y.-H., Rozan P., Lambein F., Frias J., Vidal-Valverde C. (2004). Effects of Different Germination Conditions on the Contents of Free Protein and Non-Protein Amino Acids of Commercial Legumes. Food Chem..

[38] Pumera M. (2007). Microfluidics in Amino Acid Analysis. Electrophoresis.

[39] Samtiya M., Aluko R.E., Dhewa T. (2020). Plant Food Anti-Nutritional Factors and Their Reduction Strategies: An Overview. Food Prod. Process. Nutr..

[40] Wunthunyarat W., Seo H.S., Wang Y.J. (2020). Effects of Germination Conditions on Enzyme Activities and Starch Hydrolysis of Long-Grain Brown Rice in Relation to Flour Properties and Bread Qualities. J. Food Sci..

[41] Rezaei K., Jenab E., Temelli F. (2007). Effects of Water on Enzyme Performance with an Emphasis on the Reactions in Supercritical Fluids. Crit. Rev. Biotechnol..

[42] Makinen O.E., Zannini E., Arendt E.K. (2013). Germination of Oat and Quinoa and Evaluation of the Malts as Gluten Free Baking Ingredients. Plant Foods Hum. Nutr..

[43] Lorenz K., Nyanzi F. (1989). Enzyme Activities in Quinoa (*Chenopodium quinoa*). Int. J. Food Sci. Technol..

[44] Guzmán-Ortiz F.A., Castro-Rosas J., Gómez-Aldapa C.A., Mora-Escobedo R., Rojas-León A., Rodríguez-Marín M.L., Falfán-Cortés R.N., Román-Gutiérrez A.D. (2019). Enzyme Activity During Germination of Different Cereals: A Review. Food Rev. Int..

[45] Helland M., Wicklund T., Narvhus J. (2002). Effect of Germination Time on Alpha-Amylase Production and Viscosity of Maize Porridge. Food Res. Int..

[46] Suárez-Estrella D., Bresciani A., Iametti S., Marengo M., Pagani M.A., Marti A. (2020). Effect of Sprouting on Proteins and Starch in Quinoa (*Chenopodium quinoa* Willd.). Plant Foods Hum. Nutr..

[47] Hager A.-S., Mäkinen O.E., Arendt E.K. (2014). Amylolytic Activities and Starch Reserve Mobilization During the Germination of Quinoa. Eur. Food Res. Technol..

[48] Lu S., Cik T.-T., Lii C.-y., Lai P., Chen H.-H. (2013). Effect of Amylose Content on Structure, Texture and A-Amylase Reactivity of Cooked Rice. LWT-Food Sci. Technol..

[49] Damaris R.N., Lin Z., Yang P., He D. (2019). The Rice Alpha-Amylase, Conserved Regulator of Seed Maturation and Germination. Int. J. Mol. Sci..

[50] Rosa M., Hilal M., Gonzalez J.A., Prado F.E. (2004). Changes in Soluble Carbohydrates and Related Enzymes Induced by Low Temperature During Early Developmental Stages of Quinoa (*Chenopodium quinoa*) Seedlings. J. Plant Physiol..

[51] Schlick G., Bubenheim D.L. (1993). Quinoa an Emerging New Crop with Potential for Celss. NASA Tech. Pap..

[52] Reed R.C., Bradford K.J., Khanday I. (2022). Seed Germination and Vigor: Ensuring Crop Sustainability in a Changing Climate. Heredity.

[53] del Hierro J.N., Herrera T., Fornari T., Reglero G., Martin D. (2018). The Gastrointestinal Behavior of Saponins and Its Significance for Their Bioavailability and Bioactivities. J. Funct. Foods.

[54] Ruales J., Nair B.M. (1993). Saponins, Phytic Acid, Tannins and Protease Inhibitors in Quinoa (*Chenopodium quinoa*, Willd) Seeds. Food Chem..

[55] Brady K., Ho C.-T., Rosen R.T., Sang S., Karwe M.V. (2007). Effects of Processing on the Nutraceutical Profile of Quinoa. Food Chem..

[56] Vega-GÁLvez A., San MartÍN R., Sanders M., Miranda M., Lara E. (2010). Characteristics and Mathematical Modeling of Convective Drying of Quinoa (*Chenopodium quinoa* Willd.): Influence of Temperature on the Kinetic Parameters. J. Food Process. Preserv..

[57] Guajardo-Flores D., García-Patiño M., Serna-Guerrero D., Gutiérrez-Uribe J., Serna-Saldívar S. (2012). Characterization and Quantification of Saponins and Flavonoids in Sprouts, Seed Coats and Cotyledons of Germinated Black Beans. Food Chem..

[58] Maldonado-Alvarado P., Pavón-Vargas D.J., Abarca-Robles J., Valencia-Chamorro S., Haros C.M. (2023). Effect of Germination on the Nutritional Properties, Phytic Acid Content, and Phytase Activity of Quinoa (*Chenopodium quinoa* Willd). Foods.

[59] Al-Qabba M.M., El-Mowafy M.A., Althwab S.A., Alfheeaid H.A., Aljutaily T., Barakat H. (2020). Phenolic Profile, Antioxidant Activity, and Ameliorating Efficacy of *Chenopodium quinoa* Sprouts against Ccl(4)-Induced Oxidative Stress in Rats. Nutrients.

[60] Barakat H., Spielvogel A., Hassan M., El-Desouky A., El-Mansy H., Rath F., Meyer V., Stahl U. (2010). The Antifungal Protein Afp from Aspergillus Giganteus Prevents Secondary Growth of Different Fusarium Species on Barley. Appl. Microbiol. Biotechnol..

[61] Morillo A.C., Manjarres E.H., Mora M.S. (2022). Afrosymetric Method for Quantifying Saponins in *Chenopodium quinoa* Willd. From Colombia. Braz. J. Biol..

[62] McCleary B.V., McNally M., Monaghan D., Mugford D.C. (2002). Measurement of A-Amylase Activity in White Wheat Flour, Milled Malt, and Microbial Enzyme Preparations, Using the Ceralpha Assay: Collaborative Study Barry. J. AOAC Int..

[63] Cupp-Enyard C. (2008). Sigma’s Non-Specific Protease Activity Assay—Casein as a Substrate. J. Vis. Exp..

[64] Le L., Gong X., An Q., Xiang D., Zou L., Peng L., Wu X., Tan M., Nie Z., Wu Q. (2021). Quinoa Sprouts as Potential Vegetable Source: Nutrient Composition and Functional Contents of Different Quinoa Sprout Varieties. Food Chem..

[65] Steel R.G. (1997). Principles and Procedures of Statistics A Biometrical Approach.

